# Genome sequence of a rice pest, the white-backed planthopper (*Sogatella furcifera*)

**DOI:** 10.1093/gigascience/giw004

**Published:** 2017-01-02

**Authors:** Lin Wang, Nan Tang, Xinlei Gao, Zhaoxia Chang, Liqin Zhang, Guohui Zhou, Dongyang Guo, Zhen Zeng, Wenjie Li, Ibukun A. Akinyemi, Huanming Yang, Qingfa Wu

**Affiliations:** 1 School of Life Sciences, University of Science and Technology of China, Hefei, Anhui 230027, China; 2 Guangdong Province Key Laboratory of Microbial Signals and Disease Control, College of Agriculture, South China Agricultural University, Guangzhou, Guangdong 510642, China; 3 BGI–Shenzhen, Beishan Industrial Zone, Yantian District, Shenzhen 518083, China; 4 Chinese Academy of Sciences Key Laboratory of Innate Immunity and Chronic Disease, University of Science and Technology of China, Hefei, Anhui 230027, China; 5 Hefei National Laboratory for Physical Sciences at the Microscale, Bio-X Interdisciplinary Sciences, 443 Huang-Shan Road, Hefei, Anhui 230027, China

**Keywords:** *Sogatella furcifera* genome, Genomics, Assembly, Annotation

## Abstract

**Background:** *Sogatella furcifera* is an important phloem sap-sucking and plant virus-transmitting migratory insect of rice. Because of its high reproductive potential, dispersal capability and transmission of plant viral diseases, *S. furcifera* causes considerable damage to rice grain production and has great economical and agricultural impacts. Comprehensive studies into ecological aspects and virus–host interactions of *S. furcifera* have been limited because of the lack of a well-assembled genome sequence.

**Findings:** A total of 241.3 Gb of raw reads from the whole genome of *S. furcifera* were generated by Illumina sequencing using different combinations of mate-pair and paired-end libraries from 17 insert libraries ranging between 180 bp and 40 kbp. The final genome assembly (0.72 Gb), with average N50 contig size of 70.7 kb and scaffold N50 of 1.18 Mb, covers 98.6 % of the estimated genome size of *S. furcifera*. Genome annotation, assisted by eight different developmental stages (embryos, 1^st^-5^th^ instar nymphs, 5-day-old adults and 10-day-old adults), generated 21 254 protein-coding genes, which captured 99.59 % (247/248) of core CEGMA genes and 91.7 % (2453/2675) of BUSCO genes.

**Conclusions:** We report the first assembled and annotated whole genome sequence and transcriptome of *S. furcifera*. The assembled draft genome of *S. furcifera* will be a valuable resource for ecological and virus–host interaction studies of this pest.

## Data description

The white-backed planthopper, *Sogatella furcifera* (Horvath), an r-strategy Hemiptera insect species, primarily feeds on rice plants and can migrate over long distances in the temperate and tropical regions of Asia and Australia [1]. The sucking of plant sap by *S. furcifera* reduces plant vigor, delays tillering and causes stunting, chlorosis, shriveling grains, and hopper burn, ultimately leading to rice plant death; it is responsible for the destruction of approximately 10 million hectares of rice crops annually [2]. More importantly, the planthopper transmits devastating rice viruses, including the Southern rice black-streaked dwarf virus (SRBSDV), which poses an additional threat to rice plants [3]. Insecticide misuse, cultivation of hybrid high-nutritional rice varieties, cultural and climatic factors, long-distance migration capability and robust fecundity of *S. furcifera*, together result in outbreaks of *S. furcifera* [2, 4].

## Samples and sequencing

**Figure 1.**
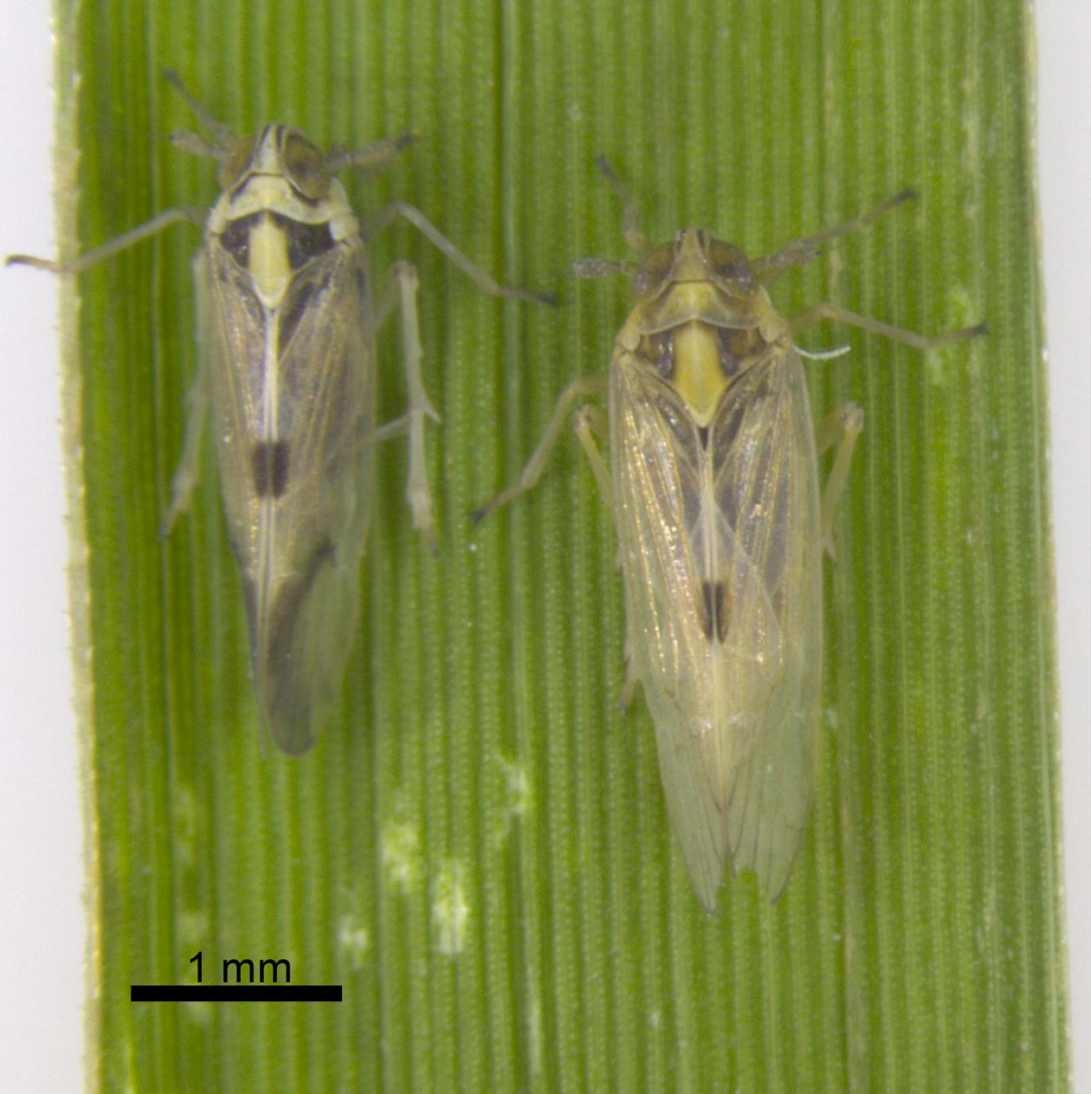
Photograph of *Sogatella furcifera* (white-backed planthopper) on a rice plant leaf. The scale bar of 1 mm is shown in the photograph.

Inbred laboratory strains of *Sogatella furcifera* (Fig. 1) originated from the University of Science and Technology of China. A laboratory colony was maintained at 26°C with 70 % humidity under a 16:8 h light/dark photoperiod for two years, spanning at least 20 generations. An inbreeding line was obtained by single pair sib-mating for 18 generations. For genome sequencing, *S. furcifera* specimens from the F6 generation were used. Seventeen DNA libraries with insert sizes ranging between 180 bp and 40 kb were constructed to perform whole genome shotgun (WGS) sequencing following the standard protocol (Illumina, San Diego, CA, USA). This generated 241.3 Gb raw sequence reads with coverage of approximately 330× (Table 1).

**Table 1.** Whole genome shotgun (WGS) reads used in sequencing of the *Sogatella furcifera* genome

	Read length	Insert size	Sequencing data	
Libraries	(bp)	(bp)	Total (G)	Coverage(x)
MiSeq	2 × 300	470	10.5	14.38
	2 × 300			
Paired-end	2 × 125	180	15.0	20.54
	2 × 125	320	13.0	17.80
	2 × 125	420	11.0	15.06
	2 × 100	350	2.4	3.28
	2 × 90	500	11.6	15.89
	2 × 125	600	8.9	12.19
	2 × 125	680	12.8	17.53
Mate-pair	2 × 125	2000	12.6	17.26
	2 × 90	3200	3.4	4.65
	2 × 125	5000	10.0	13.69
	2 × 125	8000	7.8	10.68
	2 × 125	10 000	15.9	21.78
	2 × 125	15 000	48.8	66.84
	2 × 125	20 000	24.2	33.15
	2 × 125	40 000	18.6	25.47
Total			241.3	330.54

*The estimated genome size was 0.73 Gb

For whole transcriptome sequencing, total RNA was prepared from *S. furcifella* specimens at different developmental stages. Briefly, eight different developmental stages of *S. furcifera* were washed three times with 95 % ethanol to reduce microbial contamination from the body surface. After ethanol volatilization, each sample was quickly ground into a fine powder in liquid nitrogen. Total RNA was prepared using TRIzol reagent (Invitrogen). RNA quantitation was performed by UV absorbance and its quality was further confirmed by gel electrophoresis. RNA sequencing libraries were generated using Illumina mRNA-Seq Prep Kit. In total, 75.49 Gb data comprising 603.9 million reads were generated (Table S1). For RNA-seq libraries with an insert size of 300 bp, low quality bases (Phred score< 30) were trimmed and duplicated reads were removed.

## Evaluation of genome size

Two different methods were used to estimate the genome size of *S. furcifera.* Firstly, flow cytometry was used according to a previous method [5] to estimate a genome size of about 730 Mb. Briefly, the heads of five male or female *S. furcifera* and five male *Drosophila melanogaster* (W118) specimens were ground in a tube with pestle in 200 μL labeling solution, because the genome size of *Drosophila melanogaster* has been clearly determined [6]. The mixture was filtered with a 70 μm filter(BD Falcon),then treated with RNaseA for 10 min at 37 °C and stained with 5 μg/mL propidium iodide (PI) for 2 h on ice. About 10 000 cell particles were analyzed on a flow cytometer (BD Falcon). The fluorescence value of PI was analyzed using Flowjo [7]. The mean C value (0.184 pg) and genome size (180 Mb) was calculated based on the internal *D. melanogaster* control. As a result, the genome size of a male *S. furcifera* is about 733 Mb or 0.75 pg (Fig. S1A). Experiments were conducted in triplicate. In addition, genome size was estimated based on the k-mer approach (k-mers, with k = 17) using Jellyfish [8]. In this study, approximately 56 Gb of the short-insert library sequencing data was used to generate a 17-mer depth-frequency curve (Fig. S1B). The *S. furcifera* genome size was estimated to be 735 Mb (Table S2) with 0.38 % heterozygosity as calculated by mlRho [3].

## Genome assembly and evaluation

Adapter sequences, low-quality and duplicated reads were filtered out prior to read assembly; error correction was also performed to eliminate sequencing errors. For whole-genome assembly, short reads were first assembled using SOAPdenovo2 [9], longer reads were further used for scaffolding using SSPACE [10], and finally GapCloser [11] was used to fill the gaps with short reads. Briefly, sequences derived from the short-insert libraries were decomposed into k-mers to construct the de Bruijn graph, which was simplified to allow remaining k-mers to be joined as contigs. All short-insert and large-insert libraries were mapped onto contigs for scaffold building by utilizing paired-end and mate-pair information. Paired-end and mate-pair information was subsequently applied to link contigs into scaffolds using a step-wise approach (from small to large-insert libraries). Finally, intra-scaffold gaps were filled using short-insert libraries in which one read uniquely mapped to a contig, and the other member of the pair located to a gap region. The resulting genome assembly size was 720 Mb, representing 98.6 % of the estimated genome,with a final scaffold N50 length of 1 185 287 bp and a contig N50 length of 70 730 bp. The longest contig and scaffold were 799 kb and 12.7 Mb, respectively (Table 2). In addition, reads which were mapped onto the mitochondria and Wolbachia symbiont genome sequence were extracted and assembled, and the assembled genome sizes were 16 Kb and 1.6 Mb, respectively.

**Table 2.** *Sogatella furcifera* genome assembly statistical analysis

	Contig		Scaffold	
	Size (bp)	Number	Size (bp)	Number
N90	9232	12 253	85 450	890
N80	21 047	7536	319 035	489
N70	35 405	5076	529 262	317
N60	51 883	3500	845 521	207
N50	70 730	2390	1 185 287	133
Longest	799 912	–	12 788 806	–
Total size	673 904 942	–	720 705 630	–
Total number (>10 000 bp)	602 082 273	11 792	697 471 028	2567
Total number (>100 000 bp)	258 085 068	1448	649 732 076	840

Four independent measures were used to assess the accuracy of the assembly. First, reads from paired-end libraries were mapped onto the assembly, and a greater than 10-fold effective depth was obtained across 95.55 % of the draft genome (Table S3). Second, when assembled transcripts derived from mRNA, expressed sequence tags (ESTs), and RNA-seq data from multiple developmental stages (Table S4) were mapped to the genome assembly, this revealed a transcript coverage rate of ∼98 %, suggesting that the genome was sufficiently complete for gene prediction and analysis (Tables S5–S7). Third, a core eukaryotic genes (CEG) mapping approach (CEGMA) dataset comprising 248 CEGs [12] was used to evaluate the completeness of the draft: 94.8 % (235/248) of genes were completely covered by the assembly, and 99.6 % (247/248)of genes were at least partially covered by the assembly genome (Table S8). The 5 % (13/248) incomplete genes, including three transcription factors, one ubiquitin-conjugating enzyme E2, and one mannose-6-phosphate isomerase, exhibited no obvious functional bias. Fourth, a benchmarking universal single-copy orthologs (BUSCO) dataset was used to evaluate the completeness of the draft: 91.7 % (2453/2675) of genes were covered by the assembly genome (Table S9), which was higher than 81.34% of *Nilaparvata lugens* and 81.71% of *Acyrthosiphon pisum*, respectively, because *S. furcifera, N. lugens* and *A. pisum* all belong to the Hemiptera order and were used for comparison.

## Genome characterization and repeat annotation

The *S. furcifera* genome has a G+C content of 31.6 %; compared to other hemipteran species, this is slightly higher than that of the pea aphid *A. pisum* (29.6 %) [13] but lower than that of the brown planthopper *N. lugens* (34.6 %) [14]. Homology-based and *de novo* prediction analyses were both used to identify the transposable elements (TEs) and other repetitive content in the *S. furcifera* genome. For homology-based analysis, Repbase (version 20120418) [15] was used to perform a TE search with RepeatMasker (3.3.0) [16] and the WuBlast [17] search engine. For *de novo* prediction analysis, RepeatModeler [18] was used to construct a TE library. Elements within the library were then classified using a homology search with Repbase and a Support Vector Machine (SVM) method (TEClass) [19]. A total of 44.3 % of the *S. furcifera* genome consists of tandem repeats and TEs (Table 3); lower than the brown planthopper (48.6 %) but much higher than the pea aphid (33.3 %). Class I TEs (retroelements) represent 15.3 % of the total genome assembly (9.52 % long interspersed nuclear elements (LINEs), 4.30 % long terminal repeats (LTRs) and 1.48 % short interspersed nuclear elements (SINEs)), whereas class II TEs (DNA transposons) account for 17.33 %. In addition, the *S. furcifera* genome was annotated with seven centromeric sequences [20], eight telomeric sequences[20] (Table S10) and 169 932 microsatellite regions respectively.

**Table 3.** Transposable element (TE) content of the *Sogatella furcifera* genome, derived from RepeatMasker analysis

	RepBase TEs		TE Proteins		*De novo*		Combined TEs	
	Length (bp)	% of genome	Length (bp)	% of genome	Length (bp)	% of genome	Length (bp)	% of genome
DNA	3 946 730	0.54	4 606 659	0.63	120 249 936	16.54	126 002 323	17.33
LINE	5 042 806	0.69	28 043 919	3.85	44 814 393	6.16	69 257 982	9.52
SINE	810 448	0.11	0	0	10 821 265	1.48	10 730 722	1.48
LTR	3 346 275	0.46	7 721 608	1.06	28 298 660	3.89	31 286 552	4.30
Other	975 677	0.13	317 139	0.04	23884560	328	23 167 338	3.18
Unknown	0	0.00	0	0.00	28 395 639	3.90	28 395 639	3.90
Total	14 121 936	1.94	40 689 325	5.59	256 464 453	35.27	288 840 556	39.73

Note: LINE: long interspersed nuclear element; LTR: long terminal repeat; SINE: short interspersed nuclear element.

## Annotation of coding and non-coding genes

The *S. furcifera* genome was annotated by combining homology-based methods referring to protein sequences from seven representative insects, *ab initio* gene prediction (GENSCAN [21] and AUGUSTUS [22]), and RNA-seq data from eight different developmental stages (embryos, 1^st^–5^th^ instar nymphs, 5-day-old adults and 10-day-old adults) (Table S1). For homology-based gene prediction, we aligned *Bombyx mori, D. melanogaster, Apis mellifera, A. pisum, Rhodnius prolixus, Tribolium castaneum* and *Pediculus humanus* proteins (from the Ensembl database [23]) to the *S. furcifera* genome using TblastN [24] with an E-value ≤ 1E^−5^, and then used GeneWise2.2.0 [25] for spliced alignment and prediction of gene structures. Secondly, for *ab initio* prediction, GENSCAN [21] and AUGUSTUS [22] were used to predict genes based on repeat-masked genome sequences. Short genes of <150 bp coding DNA sequences (CDS) were filtered from the resultant data sets. Thirdly, gene structure was identified using a transcriptome-based approach by mapping all RNA reads of the eight different developmental stages onto the *S. furcifera* genome using TopHat [26]. Mapping results were subsequently sorted and merged, and Cufflinks [27] was used to identify gene structures for gene annotation (Table 4). Finally, all predicted gene structures were integrated with EVidenceModeler (EVM) [28] to yield a consensus gene set containing 21 254 protein-encoding genes (Table 4), and an estimated 88 184 splice junctions [26]. Based on OrthoMCL analysis [29], the 21 254 protein-encoding genes can be assigned into 9096 single family genes, and 2963 families with 12 158 genes, indicating that *S. furcifera* has 57% duplicated genes.

**Table 4.** Characteristics of the predicted protein-coding genes in the *Sogatella furcifera* assembly

			Gene	Coding DNA sequence	Exon	Exon	Intron
Gene set		Number	length (bp)	sequence length (bp)	per gene	length (bp)	length (bp)
*De novo*	AUGUSTUS	44 600	10 406.51	1507	6.04	249	1659.97
	GENSCAN	44 160	9280.96	1106	4.25	260	1505.23
	GeneWise:						
Homolog	*Bombyx mori*	11 687	4890.78	771	2.87	268	2197.30
	*D. melanogaster*	19 671	6574.52	884	3.44	257	2328.74
	*Apis mellifera*	18 160	8437.51	1000	4.08	244	2411.35
	*Acyrthosiphon pisum*	55 250	1414.84	865	1.27	681	2032.34
	*Rhodnius prolixus*	29 842	4534.72	775	2.82	274	2054.77
	*Tribolium castaneum*	33 096	4704.18	925	2.63	351	2315.42
	*Pediculus humanus*	17 785	7868.22	986	4.01	245	2282.51
RNA-Seq		28 183	4049.83	1800	3.33	540	2504.86
EVidenceModeler		21 254	12 584.24	1577	6.47	243	2011.27

To designate gene names to each predicted protein-encoding locus, gene function information, protein motifs and domains were assigned through comparison with public databases, including Swiss-Prot [30], the Kyoto Encyclopedia of Genes and Genomes (KEGG) [31], Gene Ontology (GO) [32], TrEMBL [33] and InterPro [34]. A BLASTP [35] search of those proteomes was performed against the SwissProt [30] and TrEMBL database, with an E-value ≤ 1E^−5^. KEGG annotation was based on the KEGG Automatic Annotation Server (KAAS) [36] and GO annotation analysis was based on InterPro [34]. The 21 254 protein-encoding genes had 12 699 hits in InterPro, of which 8633 also had GO associations. In total, 59.7 %, 40.61 %, 31.26 %, 52.23 % and 68.47 % of the protein-coding gene can be assigned to known homologs in InterPro [34], GO [32], KEGG [31], SwissProt [30] and TrEMBL databases [33], respectively (Table 5). In combination, 14 990 (70.52 %) were similar to proteins from known databases. OrthoMCL analysis [29] revealed that 59.55 % and 63 % of *S. furcifera* proteins have homologs in the brown planthopper and pea aphid, respectively.

**Table 5.** Summary of functional annotation

		*Sogatella furcifera*	
		Gene number	Percent of total genes (%)
Total		21 254	–
	InterPro	12 699	59.74
	GO*	8633	40.61
Annotated	KEGG	6646	31.26
	Swiss-Prot	11 102	52.23
	TrEMBL	14 553	68.47
Annotated		14 990	70.52
Unannotated		6264	29.47

Note: Five proteins databases were chosen to assist the function prediction of genes: InterPro, GO, KEGG, Swiss-Port, and TrEMBL. The table shows numbers of genes matched in each database. *GO assignments were based on InterPro. KEGG: Kyoto Encyclopedia of Genes and Genomes, GO: Gene Ontology.

By performing a homology search across the whole genome sequence, four types of non-coding RNAs (ncRNAs) were annotated in our analysis: miRNA, tRNA, rRNA, and snRNA. tRNAscan-SE [37] was used to predict tRNA. snRNAs were predicted by alignment using BlastN and using INFERNAL (v.0.81) [38] to search against the Rfam database [39]. rRNAs were found by BlastN alignment against other insects’ rRNA as reference sequences. In total, 172 rRNAs were identified and assigned into four rRNA families (Table S11): 2256 tRNAs (including all 20 tRNA genes used for decoding standard amino acids) (Tables S11 and S12), 176 snRNAs (Table S11) comprising 37 H/ACA box RNAs and 139 snRNAs involved in splicing, as well as 382 identified miRNAs, were assigned into 110 families [40].

## Transcriptome analyses of ontogenetic development of *S. furcifera*

Low-quality bases were trimmed from RNA-seq data using a PERL script and adapters were removed using Cutadapt (version 1.3) [41]. The remaining reads were mapped against the reference sequences of rRNA and Southern rice black-streaked dwarf virus (SRBSDV) by Bowtie2 [42] using default parameters, and reads matching rRNA or SRBSDV were discarded. Filtered reads were mapped to the assembled *S. furcifera* genome with STAR [43] with the parameters: –runThreadN 8, –outFilterMultimapNmax 20, –outFilterMismatchNmax 4, –outFilterIntronMotifs: RemoveNoncanonical. Cuffdiff2 [44] was subsequently employed to calculate normalized fragments per kilobase of exon per million fragments mapped (FPKMs) based on the aligned BAM files of all RNA-seq libraries, in which the original FPKMs of each library were scaled by a library size factor computed as the median of ratios to the geometric means of original FPKMs across all libraries. Differentially expressed genes (DEGs, *P-*value<0.05 and |log_2_(fold change)|>1) in at least one comparison between any two conditions were identified with Cuffdiff2 [44] using the *blind* mode suited for a single replicate in each condition. *k*-means clustering was then performed on the gene expression profiles of DEGs with k = 8 using Gene Cluster 3.0 [45], and differential expression patterns were visualized using Java TreeView [46].

**Figure 2.**
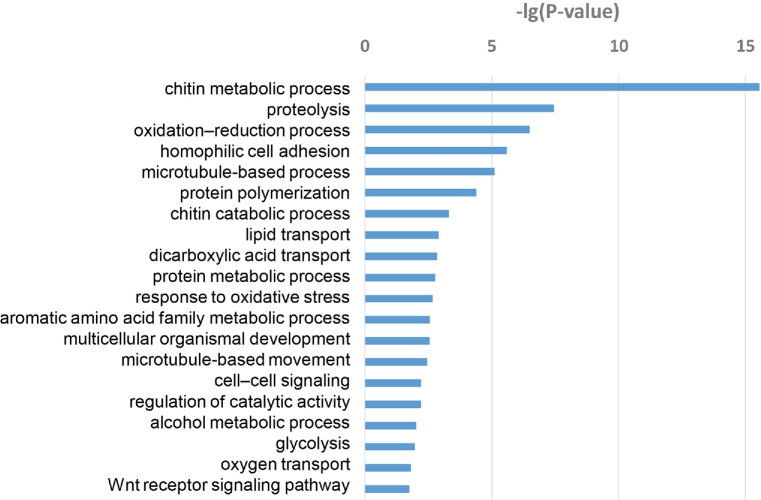
Gene ontology (GO) enrichment analysis for differentially expressed genes in eight different developmental stages of *Sogatella furcifera*. All differentially expressed genes were subjected to GO analysis – the top 20 enriched terms are shown here.

To obtain a global overview of *S. furcifera* development, the transcriptomes of eight different developmental stages, including embryo (emb), 1^st^–5^th^ instar nymphs (1in, 2in, 3in, 4in, and 5in), 5-day-old adult (5d) and 10-day-old adult (10d), were investigated using RNA-seq, with pairwise comparisons between each pair of developmental stages. A total of 4166 genes were identified as significant DEGs. Analysis of the number of genes reaching their maximum expression levels at each developmental stage showed that gene expression levels in the embryo and 10-day-old adult differed significantly from those in the other stages. GO enrichment analysis using the R package phyper [47] for all DEGs across all development stages demonstrated that these DEGs are implicated in diverse biological processes, including chitin metabolic processes, proteolysis, oxidation–reduction processes, homophilic cell adhesion and microtubule-based processes (Fig. 2).

## Expression profile clustering and expression pattern identification

*k*-means clustering on the 4166 DEGs revealed 8 different expression patterns (Fig. 3, Figs. S1–S8 and Table S13).

- A total of 864 genes were highly expressed in the embryo and at the 1^st^ instar nymph stage, with slightly higher expression levels in the embryo than in the 1^st^ instar nymph and down-regulation in later stages. GO enrichment analysis [47] associated genes in expression pattern 1 with the G protein-coupled receptor signaling pathway, ion transport, multicellular organismal development, and the Wnt receptor signaling pathway, among others. *Wnt* genes are important in embryogenesis and cell differentiation in both vertebrates and insects [48]. Wnt-4 (Sfur-175.24), Wnt-10a (Sfur-90.42) and Wnt-16 (Sfur-884.2) were clustered in pattern 1 and exhibited highest expressions during the embryo stage, suggesting that the three *Wnt* genes are involved in the embryo development of the pest.
- A total of 134 genes were classified into expression pattern 2. These genes were specifically expressed in nymph instar stages 1–5. GO enrichment analysis associated these genes with catecholamine biosynthetic processes and aromatic amino acid family metabolic processes. Catecholamine is required for insect cuticle sclerotization; catecholamine conjugates are sequestered in the hemolymph during nymphal feeding periods for later use as tanning-agent precursors in cockroaches [49]. Tyrosine 3-monooxygenase catalyzes the initial and rate-limiting step in catecholamine biosynthesis [49]. Two tyrosine 3-monooxygenase (Sfur-187.36 and Sfur-7.15), belonging to expression pattern 2, were specifically expressed in the nymph stages, suggesting that these might be responsible for catecholamine biosynthesis and have roles in the later cuticle sclerotization process.
- A total of 525 genes exhibited specifically low expression in embryos but high expression in the post-embryonic stages. These genes have been implicated in processes such as metabolism, oxidation–reduction, and transmembrane transport. Several genes involved in fatty acid synthesis, including fatty acid synthase (Sfur-509.5, Sfur-698.4 and Sfur-72.518) and long-chain-fatty-acid-CoA ligase (Sfur-188.12 and Sfur-215.3), belonged to pattern 3 and were highly expressed in the nymph and adult stages.
- A total of 591 genes exhibited relatively higher expression levels from the 2^nd^ to the 5^th^ instar nymph, but were slightly down-regulated in the 4^th^ instar nymph. These genes are enriched in nucleosome assembly, reciprocal meiotic recombination, DNA catabolism, and chitin metabolic processes.
- A total of 523 genes were specifically expressed in the 3^rd^ and 4^th^ instar nymphs, including genes participating in microtubule-based movement, glycolysis, glycerol metabolic processes, and positive regulation of apoptotic processes. Glycolysis can be suppressed during molting to direct a feeding, growing larva to convert to the immobile non-feeding stage [50]. Several genes participating in glycolysis, including fructose-bisphosphate aldolase (Sfur-330.7) and pyruvate kinase (Sfur-409.10 and Sfur-78.49), belonged to pattern 5; these were highly expressed in the 3^rd^ and 4^th^ instar nymphs and down-regulated in the 5^th^ instar nymph.
- A total of 218 genes were highly expressed from the 3^rd^ instar nymph to the 5-day-old adult, but the expression levels were higher in the 3^rd^ and 4^th^ instar nymphs than in the 5^th^ instar nymphs and 5-day-old adults. These genes are related to processes including protein metabolism, microtubule-based processes, and negative regulation of biosynthesis.
- A total of 609 genes were highly expressed from the embryo to the 5^th^ instar nymph, then down-regulated in adults. These genes were enriched in biological processes including chitin metabolism and catabolism, alcohol metabolism, steroid hormone mediated signaling pathways, and ecdysis, chitin-based cuticles. Many genes belonging to pattern 7 were involved in chitin metabolic processes and were highly expressed from embryo to the 5^th^ instar nymphs; for example,endochitinase (Sfur-47.4), chitinase (Sfur-18.277, Sfur-203.28 and Sfur-453.6), chitotriosidase (Sfur-97.70), and peritrophin (Sfur-105.51, Sfur-84.51 and Sfur-203.26). Chitin is the main component of insect exoskeleton and peritrophic matrix. Chitin metabolism is coupled with insect growth and development. Because the exoskeleton and peritrophic matrix are regularly replaced and renewed during insect growth, chitin metabolic process should be active throughout the embryo and nymph stages until growing and melting stages are completed. The crucial role of chitin in insect development and survival has led to chitin-related genes being targeted for the development of pest control strategies [51].
- A total of 702 genes were specifically expressed in the 5-day-old and 10-day-old adults. These genes are involved in processes such as lipid transport, DNA repair, cell–cell signaling, and microtubule-based movement. Vitellogenin, a member of the lipid transport protein family, is a precursor of egg yolk, which is specifically expressed in females and provides the essential nutrients required for egg development [52]. Vitellogenin (Sfur-20.301, Sfur-20.304, Sfur-3160.1, Sfur-496.9, Sfur-15.299 and Sfur-20.302) belonged to pattern 8 and was highly expressed in 5-day-old and 10-day-old adults, suggesting that they are essential for the *S. furcifera* reproduction process.

**Figure 3.**
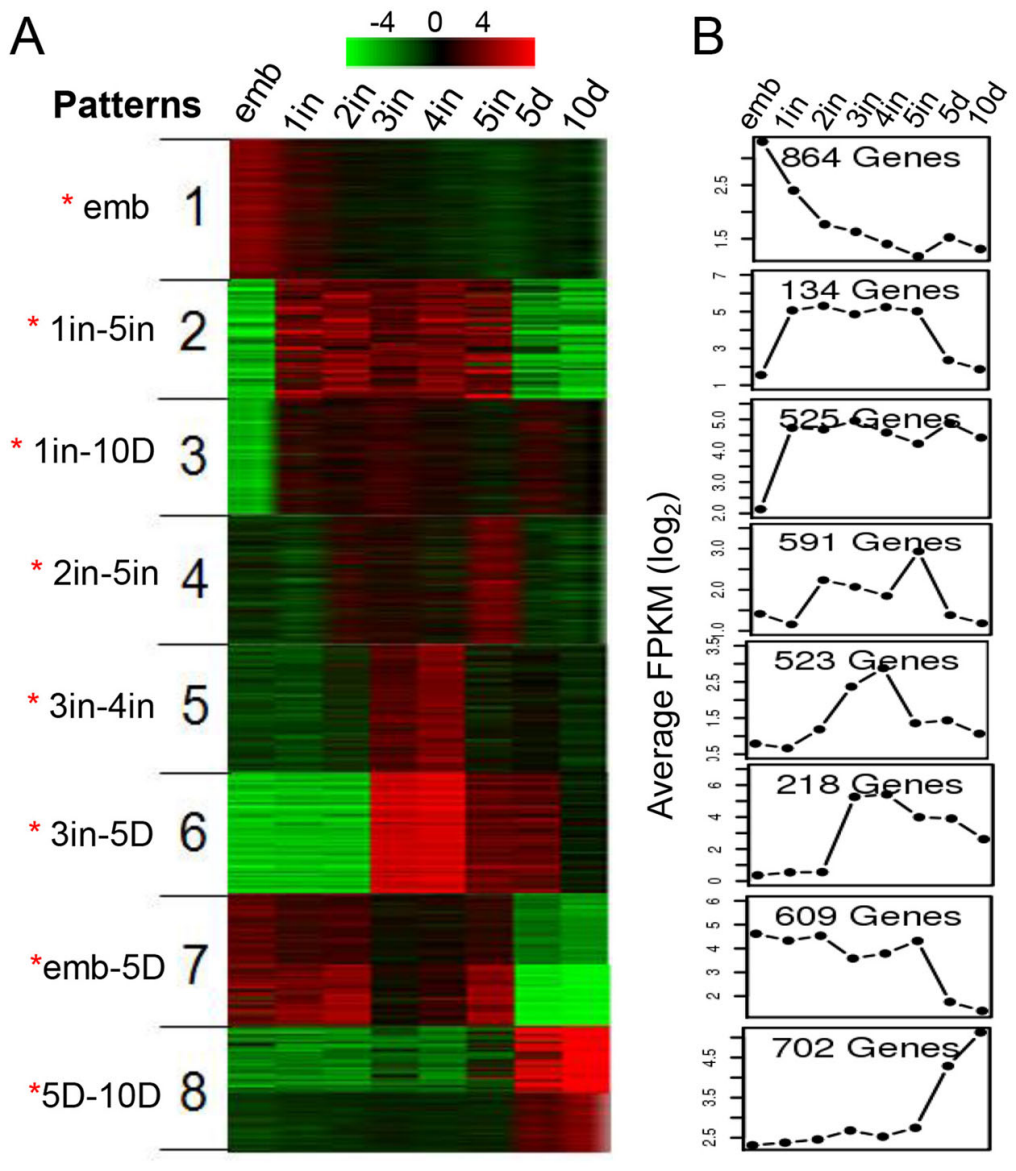
*k*-means clustering for differentially expressed genes and expression patterns. **(A)** Eight expression patterns are shown on the left. Heat map shows the relative expression levels of each transcript (rows) in each sample (column). Normalized fragments per kilobase of exon per million fragments (FPKMs) calculated by Cuffdiff2 were log_2_-transformed and then median-centered by transcript. Heatmap was drawn based on clustering results. Red color represents higher expression; green represents lower expression. Note: red asterisks (*) on the left side of the figure indicate that expression at the corresponding stage is higher than the average expression level. Abbreviations: emb: embryo; 1in: 1^st^ instar nymph; 2in: 2^nd^ instar nymph; 3in: 3^rd^ instar nymph; 4in: 4^th^ instar nymph; 5in: 5^th^ instar nymph; 5d: 5-day-old adult; 10d: 10-day-old adult. **(B)** The average of log_2_-transformed FPKM corresponding genes in each pattern.

## Conclusions

The lack of a genome sequence for *S. furcifera* has hindered comprehensive studies of the life pattern and adaptive features that have made it a successful insect pest. We present the first annotated genome sequence of *S. furcifera* and data on the corresponding protein-coding genes that will aid future detailed studies into the insect's biology and virus–host interactions.

## Availability of supporting data

Raw and transcriptomic data is available via NCBI bioproject PRJNA331022. Further data and scripts supporting the results of this article are available in the *GigaScience* GigaDB repository [53].

## Additional file

Supplementary data are available at *GIGSCI* online.

**Additional file 1: Table S1**. Transcriptome sequencing data statistics.

**Additional file 2: Table S2**. Estimation of *Sogatella furcifera* genome size using k-mer analysis.

**Additional file 3: Table S3**. Alignment information of short read mapping to the genome.

**Additional file 4: Table S4**. RNA-seq datasets used in this study.

**Additional file 5: Table S5**. Assessment of genome coverage by *Sogatella furcifera* transcripts assembled from mutiple developmental stages reads.

**Additional file 6: Table S6**. Assessment of genome coverage by *Sogatella furcifera* expressed sequence tags.

**Additional file 7: Table S7**. Assessment of genome coverage by *Sogatella furcifera* assembled transcripts.

**Additional file 8: Table S8**. Genome assembly completeness evaluated on 248 core eukaryotic genes.

**Additional file 9: Table S9**. Genome assembly completeness evaluated using benchmarking universal single-copy orthologs (BUSCO).

**Additional file 10: Table S10**. Centromeric and telomeric DNA in *Sogatella furcifera.*

**Additional file 11: Table S11**. Non-protein-coding genes in *Sogatella furcifera.*

**Additional file 12: Table S12**. Isotype and anticodon count distribution of tRNA.

**Additional file 13: Table S13**. Gene numbers in each expression pattern.

**Additional file 14: Figure S1**. Estimation of *Sogatella furcifera* genome size based on flow cytometry and 17-mer statistics. (A) Estimation of *Sogatella furcifera* genome size based on flow cytometry. Comparing with the *Drosophila melanogaster* (*D. mel*) genome, the C value of *S furcifera* (*S. fur*) genome was estimated to be 0.75 pg. The genome size of *S. furcifera* was estimated to be 733 Mb. (B) Estimation of *S. furcifera* genome size based on 17-mer statistics. In total 56.76 Gb of short (<1 kb) paired-end genome sequencing reads were used to generate the 17-mer sequences. The genome size of *S. furcifera* was estimated to be 730 Mb based on the formula: 17-mer number/17-mer depth.

**Additional file 15: Figure S2**. Gene Ontology enrichment analysis for genes in expression pattern 1.

**Additional file 16: Figure S3**. Gene Ontology enrichment analysis for genes in expression pattern 2.

**Additional file 17: Figure S4**. Gene Ontology enrichment analysis for genes in expression pattern 3. The top 20 enriched terms are shown.

**Additional file 18: Figure S5**. Gene Ontology enrichment analysis for genes in expression pattern 4.

**Additional file 19: Figure S6**. Gene Ontology enrichment analysis for genes in expression pattern 5.

**Additional file 20: Figure S7**. Gene Ontology enrichment analysis for genes in expression pattern 6.

**Additional file 21: Figure S8**. Gene Ontology enrichment analysis for genes in expression pattern 7.

**Additional file 22: Figure S9**. Gene Ontology enrichment analysis for genes in expression pattern 8. The top 20 enriched terms are shown.

GIGA-D-16-00057_Original_Submission.pdf

GIGA-D-16-00057_Revision_1.pdf

GIGA-D-16-00057_Revision_2.pdf

Response_to_Reviewer_Comments_Original_Submission.pdf

Response_to_Reviewer_Comments_Revision_1.pdf

Reviewer_1_Report_(Original_Submission).pdf

Reviewer_1_Report_(Revision_1).pdf

Reviewer_2_Report_(Original_Submission).pdf

Reviewer_2_Report_(Revision_1).pdf

Supplemental material **Additional file 1: Table S1**. Transcriptome sequencing data statistics.
**Additional file 2: Table S2**. Estimation of *Sogatella furcifera* genome size using k-mer analysis.
**Additional file 3: Table S3**. Alignment information of short read mapping to the genome.
**Additional file 4: Table S4**. RNA-seq datasets used in this study.
**Additional file 5: Table S5**. Assessment of genome coverage by *Sogatella furcifera* transcripts assembled from mutiple developmental stages reads.
**Additional file 6: Table S6**. Assessment of genome coverage by *Sogatella furcifera* expressed sequence tags.
**Additional file 7: Table S7**. Assessment of genome coverage by *Sogatella furcifera* assembled transcripts.
**Additional file 8: Table S8**. Genome assembly completeness evaluated on 248 core eukaryotic genes.
**Additional file 9: Table S9**. Genome assembly completeness evaluated using benchmarking universal single-copy orthologs (BUSCO).
**Additional file 10: Table S10**. Centromeric and telomeric DNA in *Sogatella furcifera.*
**Additional file 11: Table S11**. Non-protein-coding genes in *Sogatella furcifera.*
**Additional file 12: Table S12**. Isotype and anticodon count distribution of tRNA.
**Additional file 13: Table S13**. Gene numbers in each expression pattern.
**Additional file 14: Figure S1**. Estimation of *Sogatella furcifera* genome size based on flow cytometry and 17-mer statistics. (A) Estimation of *Sogatella furcifera* genome size based on flow cytometry. Comparing with the *Drosophila melanogaster* (*D. mel*) genome, the C value of *S furcifera* (*S. fur*) genome was estimated to be 0.75 pg. The genome size of *S. furcifera* was estimated to be 733 Mb. (B) Estimation of *S. furcifera* genome size based on 17-mer statistics. In total 56.76 Gb of short (<1 kb) paired-end genome sequencing reads were used to generate the 17-mer sequences. The genome size of *S. furcifera* was estimated to be 730 Mb based on the formula: 17-mer number/17-mer depth.
**Additional file 15: Figure S2**. Gene Ontology enrichment analysis for genes in expression pattern 1.
**Additional file 16: Figure S3**. Gene Ontology enrichment analysis for genes in expression pattern 2.
**Additional file 17: Figure S4**. Gene Ontology enrichment analysis for genes in expression pattern 3. The top 20 enriched terms are shown.
**Additional file 18: Figure S5**. Gene Ontology enrichment analysis for genes in expression pattern 4.
**Additional file 19: Figure S6**. Gene Ontology enrichment analysis for genes in expression pattern 5.
**Additional file 20: Figure S7**. Gene Ontology enrichment analysis for genes in expression pattern 6.
**Additional file 21: Figure S8**. Gene Ontology enrichment analysis for genes in expression pattern 7.
**Additional file 22: Figure S9**. Gene Ontology enrichment analysis for genes in expression pattern 8. The top 20 enriched terms are shown.

## Abbreviations

BUSCO: benchmarking universal single-copy orthologs; CDS: coding DNA sequence; CEG: core eukaryotic gene; CEGMA: core eukaryotic gene mapping approach; DEG: differentially expressed gene; EST: expressed sequence tag; EVM: EVidenceModeler; FPKM: fragments per kilobase of exon per million fragments; KEGG: Kyoto Encyclopedia of Genes and Genomes; GO: Gene Ontology; LINE: long interspersed nuclear element; LTR: long terminal repeat; miRNA: microRNA; ncRNA: non-coding RNA; PI: propidium iodide; snRNA: small nuclear RNA; TE: transposable element; WGS: whole genome shotgun; SINE: short interspersed nuclear element; SRBSDV: Southern rice black-streaked dwarf virus; SVM: support vector machine.

## Competing interests

The authors declare they have no competing interests.

## Funding

This work was supported by the Strategic Priority Research Program of the Chinese Academy of Sciences (Grant XDB11040400), the Ministry of Science and Technology of China (Grant 2014CB138405), and the National Natural Science Foundation of China (Grants 31571305, 91231110, and 31272011). The funders played no role in the study design, data collection and analysis, decision to publish, or preparation of the manuscript.

## Authors’ contributions

QW conceived the study and designed the experiments. NT, LZ, GZ, DG, LW and WL conducted the sample preparation, DNA/RNA isolation for sequencing and library construction. LW and QW performed the genome assembly, annotation, evaluation, and comparative genomics analysis and evolution studies. LW, XG, ZC, ZZ and IA conducted the transcriptome assembly and gene differential expression analysis. LW, HY and QW drafted and revised the manuscript and supplementary information. All authors read and approved the final manuscript.

## References

[1] Mun JH, Song YH, Heong KL, Roderick GK Genetic variation among Asian populations of rice planthoppers, Nilaparvata lugens and Sogatella furcifera (Hemiptera: Delphacidae): mitochondrial DNA sequences. Bull Entomol Res. 1999;89:245–53.

[2] Cheng J Rice planthopper problems and relevant causes in China. Planthoppers: New Threats to the Sustainability of Intensive Rice Production Systems in Asia. 2009;157–78.

[3] Zhou G, Wen J, Cai D, Li P, Xu D, Zhang S Southern rice black-streaked dwarf virus: A new proposed Fijivirus species in the family Reoviridae. Chin Sci Bull. 2008;53:3677–85.

[4] Zhai Y, Zhang J, Sun Z, Dong X, He Y, Kang K Proteomic and transcriptomic analyses of fecundity in the brown planthopper Nilaparvata lugens (Stal). Journal of Proteome Research. 2013;12:5199–212. 24083549 10.1021/pr400561c

[5] Nardon C, Weiss M, Vieira C, Biémont C Variation of the genome size estimate with environmental conditions in Drosophila melanogaster. Cytometry Part A. 2003;55:43–49. 10.1002/cyto.a.10061 12938187

[6] Ellis LL, Huang W, Quinn AM, Ahuja A, Alfrejd B, Gomez FE Intrapopulation genome size variation in D. melanogaster reflects life history variation and plasticity. PLoS Genet. 2014;10:e1004522. 25057905 10.1371/journal.pgen.1004522 PMC4109859

[7] Baumgarth N, Roederer M A practical approach to multicolor flow cytometry for immunophenotyping. J Immunol Methods. 2000;243:77–97. 10986408 10.1016/s0022-1759(00)00229-5

[8] Marcais G, Kingsford C A fast, lock-free approach for efficient parallel counting of occurrences of k-mers. Bioinformatics. 2011;27:764–70. 21217122 10.1093/bioinformatics/btr011 PMC3051319

[9] Luo RB, Liu BH, Xie YL, Li ZY, Huang WH, Yuan JY SOAPdenovo2: an empirically improved memory-efficient short-read de novo assembler. GigaScience 2012;1:18. 23587118 10.1186/2047-217X-1-18 PMC3626529

[10] Boetzer M, Henkel CV, Jansen HJ, Butler D, Pirovano W Scaffolding pre-assembled contigs using SSPACE. Bioinformatics. 2011;27:578–79. 21149342 10.1093/bioinformatics/btq683

[11] Li RQ, Zhu HM, Ruan J, Qian WB, Fang XD, Shi ZB De novo assembly of human genomes with massively parallel short read sequencing. Genome Res. 2010;20:265–72. 20019144 10.1101/gr.097261.109 PMC2813482

[12] Parra G, Bradnam K, Ning Z, Keane T, Korf I Assessing the gene space in draft genomes. Nucleic Acids Res. 2009;37:289–97. 19042974 10.1093/nar/gkn916 PMC2615622

[13] International Aphid Genomics C: Genome sequence of the pea aphid Acyrthosiphon pisum. PLoS Biol. 2010;8:e1000313. 20186266 10.1371/journal.pbio.1000313 PMC2826372

[14] Xue J, Zhou X, Zhang CX, Yu LL, Fan HW, Wang Z Genomes of the rice pest brown planthopper and its endosymbionts reveal complex complementary contributions for host adaptation. Genome Biol. 2014;15:521. 25609551 10.1186/s13059-014-0521-0 PMC4269174

[15] Jurka J, Kapitonov VV, Pavlicek A, Klonowski P, Kohany O, Walichiewicz J Repbase update, a database of eukaryotic repetitive elements. Cytogenetic Genome Res. 2005;110:462–67. 10.1159/000084979 16093699

[16] Smit AF, Hubley R, Green P RepeatMasker Open-3.0. 1996 http://www.repeatmasker.org/cgi-bin/WEBRepeatMasker.

[17] Gish W Wu-blast. 1996 http://www.ebi.ac.uk/Tools/webser-vices/services/sss/wu_blast_soap.

[18] Smit AF, Hubley R RepeatModeler Open-1.0. Repeat Masker Website. 2010 http://www.repeatmasker.org/Repeat-Modeler.html.

[19] Abrusan G, Grundmann N, DeMester L, Makalowski W TEclass-a tool for automated classification of unknown eukaryotic transposable elements. Bioinformatics. 2009;25:1329–30. 19349283 10.1093/bioinformatics/btp084

[20] Okazaki S, Tsuchida K, Maekawa H, Ishikawa H, Fujiwara H Identification of a pentanucleotide telomeric sequence, (TTAGG) n, in the silkworm Bombyx mori and in other insects. Mol Cell Biol. 1993;13:1424–32. 8441388 10.1128/mcb.13.3.1424-1432.1993 PMC359452

[21] Burge C, Karlin S Prediction of complete gene structures in human genomic DNA. J Mol Biol. 1997;268:78–94. 9149143 10.1006/jmbi.1997.0951

[22] Stanke M, Morgenstern B AUGUSTUS: a web server for gene prediction in eukaryotes that allows user-defined constraints. Nucleic Acids Res. 2005;33:W465–67. 15980513 10.1093/nar/gki458 PMC1160219

[23] Kersey PJ, Allen JE, Armean I, Boddu S, Bolt BJ, Carvalho-Silva D Ensembl Genomes 2016: more genomes, more complexity. Nucleic Acids Res. 2016;44:D574–80. 26578574 10.1093/nar/gkv1209 PMC4702859

[24] Gertz EM, Yu Y-K, Agarwala R, Schäffer AA, Altschul SF Composition-based statistics and translated nucleotide searches: improving the TBLASTN module of BLAST. BMC Biol. 2006;4:1. 17156431 10.1186/1741-7007-4-41 PMC1779365

[25] Birney E, Clamp M, Durbin R GeneWise and genomewise. Genome Res. 2004;14:988–95. 15123596 10.1101/gr.1865504 PMC479130

[26] Trapnell C, Pachter L, Salzberg SL TopHat: discovering splice junctions with RNA-Seq. Bioinformatics. 2009;25:1105–11. 19289445 10.1093/bioinformatics/btp120 PMC2672628

[27] Trapnell C, Williams BA, Pertea G, Mortazavi A, Kwan G, van Baren MJ Transcript assembly and quantification by RNA-Seq reveals unannotated transcripts and isoform switching during cell differentiation. Nat biotechnol. 2010;28:511–U174. 20436464 10.1038/nbt.1621 PMC3146043

[28] Haas BJ, Salzberg SL, Zhu W, Pertea M, Allen JE, Orvis J Automated eukaryotic gene structure annotation using EVidenceModeler and the program to assemble spliced alignments. Genome Biol. 2008;9:R7. 18190707 10.1186/gb-2008-9-1-r7 PMC2395244

[29] Li L, Stoeckert CJ, Roos DS OrthoMCL: Identification of ortholog groups for eukaryotic genomes. Genome Res. 2003;13:2178–89. 12952885 10.1101/gr.1224503 PMC403725

[30] Yip YL, Scheib H, Diemand AV, Gattiker A, Famiglietti LM The Swiss-Prot variant page and the ModSNP database: A resource for sequence and structure information on human protein variants. Hum Mutat. 2004;23:464–70. 15108278 10.1002/humu.20021

[31] Kanehisa M, Goto S KEGG: kyoto encyclopedia of genes and genomes. Nucleic Acids Res. 2000;28:27–30. 10592173 10.1093/nar/28.1.27 PMC102409

[32] Consortium GO The Gene Ontology (GO) database and informatics resource. Nucleic Acids Res. 2004;32:D258–61. 14681407 10.1093/nar/gkh036 PMC308770

[33] O’Donovan C, Martin MJ, Gattiker A, Gasteiger E, Bairoch A, Apweiler R High-quality protein knowledge resource: SWISS-PROT and TrEMBL. Brief bioinform. 2002;3:275–84. 12230036 10.1093/bib/3.3.275

[34] Hunter S, Apweiler R, Attwood TK, Bairoch A, Bateman A, Binns D InterPro: the integrative protein signature database. Nucleic Acids Res. 2009;37:D211–15. 18940856 10.1093/nar/gkn785 PMC2686546

[35] Altschul SF, Gish W, Miller W, Myers EW, Lipman DJ Basic local alignment search tool. J Mol Biol. 1990;215:403–10. 2231712 10.1016/S0022-2836(05)80360-2

[36] Moriya Y, Itoh M, Okuda S, Yoshizawa AC, Kanehisa M KAAS: an automatic genome annotation and pathway reconstruction server. Nucleic Acids Res. 2007;35:W182–5. 17526522 10.1093/nar/gkm321 PMC1933193

[37] Lowe TM, Eddy SR tRNAscan-SE: A program for improved detection of transfer RNA genes in genomic sequence. Nucleic Acids Res. 1997;25:955–64. 9023104 10.1093/nar/25.5.955 PMC146525

[38] Nawrocki EP, Eddy SR Infernal 1.1: 100-fold faster RNA homology searches. Bioinformatics. 2013;29:2933–5. 24008419 10.1093/bioinformatics/btt509 PMC3810854

[39] Griffiths-Jones S, Bateman A, Marshall M, Khanna A, Eddy SR Rfam: an RNA family database. Nucleic Acids Res. 2003;31:439–41. 12520045 10.1093/nar/gkg006 PMC165453

[40] Chang ZX, Tang N, Wang L, Zhang LQ, Akinyemi IA, Wu QF Identification and characterization of microRNAs in the white-backed planthopper, Sogatella furcifera. Insect Sci. 2016;23:452–68. 27060479 10.1111/1744-7917.12343

[41] Martin M. Cutadapt removes adapter sequences from high-throughput sequencing reads. EMBnet journal. 2011;17:10–2.

[42] Langmead B, Salzberg SL Fast gapped-read alignment with Bowtie 2. Nat methods. 2012;9:357–9. 22388286 10.1038/nmeth.1923 PMC3322381

[43] Dobin A, Davis CA, Schlesinger F, Drenkow J, Zaleski C, Jha S STAR: ultrafast universal RNA-seq aligner. Bioinformatics. 2013;29:15–21. 23104886 10.1093/bioinformatics/bts635 PMC3530905

[44] Trapnell C, Hendrickson DG, Sauvageau M, Goff L, Rinn JL, Pachter L Differential analysis of gene regulation at transcript resolution with RNA-seq. Nat biotechnol. 2013;31:46–53. 23222703 10.1038/nbt.2450 PMC3869392

[45] De Hoon MJ, Imoto S, Nolan J, Miyano S Open source clustering software. Bioinformatics. 2004;20:1453–54. 14871861 10.1093/bioinformatics/bth078

[46] Page RD TreeView. 2001, Glasgow, UK: Glasgow University.

[47] Johnson NL, Kemp AW, Kotz S Univariate discrete distributions. 2005, John Wiley & Sons.

[48] Holstein TW The evolution of the Wnt pathway. CSH Perspect Bio. 2012;4:a007922. 10.1101/cshperspect.a007922 PMC3385961 22751150

[49] Hopkins TL, Kramer KJ Insect Cuticle Sclerotization. Ann Rev Entomol. 1992;37:273–302.

[50] Tian L, Guo EE, Wang S, Liu SM, Jiang RJ, Cao Y Developmental Regulation of Glycolysis by 20-hydroxyecdysone and Juvenile Hormone in Fat Body Tissues of the Silkworm, Bombyx mori. J Mol Cell Biol. 2010;2:255–63. 20729248 10.1093/jmcb/mjq020

[51] Zhu KY, Merzendorfer H, Zhang WQ, Zhang JZ, Muthukrishnan S Biosynthesis, turnover, and functions of chitin in insects. Ann Rev Entomol. 2016;61:177–96. 26982439 10.1146/annurev-ento-010715-023933

[52] Hansen IA, Attardo GM, Rodriguez SD, Drake LL Four-way regulation of mosquito yolk protein precursor genes by juvenile hormone-, ecdysone-, nutrient-, and insulin-like peptide signaling pathways. Front Physiol. 2014;5. 10.3389/fphys.2014.00103 PMC3960487 24688471

[53] Wang L, Tang N, Gao X, Chang Z, Zhang L, Zhou G (2016): Supporting data for “Genome sequence of a rice pest, the white-backed planthopper (*Sogatella furcifera*)”. GigaScience Database. http://dx.doi.org/10.5524/100255. 10.1093/gigascience/giw004 PMC5437944 28369349

