# Erratum to: Genome sequence of a rice pest, the white-backed planthopper (*Sogatella furcifera*)

**DOI:** 10.1093/gigascience/gix068

**Published:** 2017-10-06

**Authors:** Lin Wang, Nan Tang, Xinlei Gao, Zhaoxia Chang, Liqin Zhang, Guohui Zhou, Dongyang Guo, Zhen Zeng, Wenjie Li, Ibukun A. Akinyemi, Huanming Yang, Qingfa Wu

**Correction**

In the final publication of “Genome sequence of a rice pest, the white-backed planthopper *(Sogatella furcifera*),” by Lin Wang et al., the listed accepted date was incorrect, and the article did not include the revised date. The accepted date has been corrected and the revised date added. The corrected manuscript can be found online at https://academic.oup.com/gigascience/article/6/1/1/2865203/Genome-sequence-of-a-rice-pest-the-white-backed?searchresult=1.

The Publisher regrets these errors.

